# The Story of the Insane from Year to Year

**Published:** 1896-08-08

**Authors:** 


### THE STORY OF THE INSANE FROM YEAR TO YEAR.

Ninth Series.

### THE CITY OF LONDON ASYLUM.

On January 1st this asylum contained 473 patients, and during the year the numbers were practically stationary. There were 110 admissions; 24 discharged recovered; 53 discharged relieved; and 27 deaths. The recoveries stand at 28·23 per cent. on the admissions, and the deaths at 5·76 on the average number resident. Both of these percentages are low. Ten of the deaths were caused by spinal and cerebral diseases, while there were no deaths from consumption. Hereditary influence accounts for 22 of the admissions, drink for 17, religion for 5, and love for 3. This is one of our public asylums which does useful work in admitting private patients, and at the close of 1895 there were 61 of these in residence. Dr. White says the accommodation is highly appreciated, and that during the year he had to refuse many patients for want of room. We know by the number of inquiries we receive on the subject that it is extremely difficult for the friends of patients to find accommodation for their insane relatives at moderate cost. The charge at the City of London is £1 1s. a week, but even this is more than many people can afford, and we hope Dr. White may see his way to make a considerable reduction in this rate, at least for chronic cases. The visitors say they have pleasure in recording their appreciation of the zealous and efficient manner in which the medical superintendent has discharged his duties. The rate is 10s. 5d. per head per week.

### THE BUTLER HOSPITAL FOR THE INSANE, PROVIDENCE, RHODE ISLAND.

This is a charitable institution somewhat resembling our own lunatic hospitals, and it this year issues its fifty-second annual report, from which we learn that the number of patients has nearly doubled during its period of existence. Last year there were 79 admissions, 14 were discharged recovered, 19 relieved, and 20 died. When discussing the popular ideas concerning the curableness of insanity, Dr. Gorton shows that mere statistics are valueless when comparing the recovery rate of one institution with another. "It would seem tolerably clear," he says, "that statements of results of treatment based simply upon the consideration of the number of patients admitted to an institution, or to a series of institutions, and upon the number of discharged as recovering or otherwise, can be of relatively small value as criteria of criticism." This is certainly true of the comparisons made between one asylum and another, and if such comparisons be made between lunatic asylums and general hospitals, the conclusions based on them would be not only useless but absurd. Although we do not recommend the cumbrous mass of statistical tables which accompanies many of our asylum reports we think the medical superintendent reports might do a little more that way. The average cost per week per head is 11 dollars and 60 cents.

### THE DOWN DISTRICT ASYLUM.

At the beginning of the year 501 patients were in residence, and this is 80 in excess of the number for which proper accommodation is provided. There were 138 admissions, 66 recoveries, and 27 deaths. The percentage of recoveries is unusually high, standing at 47·82, while the percentage of deaths is low, being only 5·22. Intemperance in drink caused the disease in 14 of the year's admissions, and hereditary influence in 49. Cerebral diseases were the causes of death in five instances, and consumption in six. Dr. Nolan does not think that insanity is increasing, and he thinks the increase in the number of the registered insane is owing to the benefit which patients derive from asylum treatment. In fact, confidence in the management of asylums has taken the place of the fear and dread in which these places were at one time held; and as Dr. Nolan puts it, "the increase is in our enlightenment, and not in the disease, and our greatest misfortune is that we are called on to pay the pecuniary penalty of our progress." We know it would be difficult to prove, but we are inclined to think that after making all allowances there is a slight increase in the amount of insanity. The average cost per head per annum is £20 15s.

